# Book Review: Scientific Method: How Science Works, Fails to Work and Pretends to Work

**DOI:** 10.3389/fpsyg.2018.02260

**Published:** 2018-11-20

**Authors:** Micah Amd

**Affiliations:** ^1^Department of Psychology, Federal University of Sao Carlos, Sao Carlos, Brazil; ^2^Department of Neurology, Montreal Neurological Institute, Montreal, QC, Canada

**Keywords:** research method, psychology - study and teaching, learning, social science, economics

This book aims to disinfect egregious practices in social science by illustrating errors commonly made by many social scientists, including some with Nobel prizes. Professor John Staddon (JS) is one of those rare individuals held in equally high regard for his seminal works in experimental and theoretical Psychology (Staddon, 2001), and readers stand to greatly benefit from the litany of suggestions borne of JS's experience. One notable highlight is the author's long-standing criticism of static (time-independent) theorizing in Psychology, which generally requires some “executive control” system to initiate operations (cf., Bandura's self-system—p. 63). The author suggests that psychological scientists incorporate *time* as a constitutive element within their explanatory models, fortifying his thesis through examples of complex behavioral systems, such as a child's expectation of punishment following a display of aggression (p. 66), without reference to some intervening all-knowing homunculus. Professor JS is among the forerunners of the silent behavioristic renaissance (Staddon, 2014) and readers may be surprised to know how this once maligned science of “muscle twitches” and “glandular squirts” (Bower, 2014) evolved into a compelling alternative to buttress against the excessive “surplus meaning” underlying information processing approaches in Psychology (Amsel, 1992).

The book consists of eight brief and informative chapters, with Chapters 1 through 3 showcasing to the reader methodological oversights common to scientific conduct. The remaining chapters focus on problems more specific to a given area in social science. Chapter 1 eloquently posits the processes of induction (approximating some rule behind a phenomenon's occurrence) and deduction (generating causal predictions, or hypotheses, from the earlier approximated rule) as equally necessary for scientific understanding, countering claims by those who imply the utility of one process over another (e.g., Hayes et al., 2001, p. 144). This opening salvo will re-orient social scientists who have been led astray through misinformation about what constitutes scientific understanding.

Chapters 2 and 3 go on to detail specifics regarding experimental methodology and the disadvantages from relying exclusively on null hypothesis testing, which can include (for example) not considering how differences in *a priori* information affects the probability distributions of the variables under investigation, and consequently, using the same null hypothesis and alpha criteria across all research questions. Indeed, the *p* < 0.05 significance threshold employed across much of social science was originally meant to discriminate across crop yields (pp. 33–34), not for disentangling behaviors of complex systems! Finally, it is worth restating that rejecting a null statement about the relationship between groups of subjects tells us nothing about any *specific* relationship between the groups compared. Alongside recommending more rigorous statistics, including the reporting of effect sizes, more conservative significance thresholds (*p* < 0.001), and the use of Bayesian priors, the author reminds us of the merits underlying the neglected, but still powerful, single-subject approach in experimental psychology. First championed by B. F. Skinner, single-subject approaches can verify cause-and-effect relations across individuals with a resolution group comparison cannot match. Keeping in mind that many psychologically interesting topics are defined, and must correspondingly be explained, at the group level (e.g., gender identity, or political affiliation), JS's exposition remains invaluable as it showcases to readers how sound psychological analyses need not be restricted to large-scale group comparisons and classical frequentist approaches.

Most methodological errors, at least in social science, should be detectable by the reader if the lessons in the first three chapters were carefully attended to, but JS's suggestions go beyond mere technical refinements. Over the remainder of the text, JS focuses on the thornier issue of confused terminologies in social science. An excellent example is provided in the discussion on “heredity” and “intelligence” in Chapter 4, where JS points out that most individuals outside the intelligence research community assume researchers talk about *genetic* heritability when what researchers typically imply is *statistical* heritability. Regarding the former, JS argues we know little about how parental genes cause a child's brain, let alone her behavior, whereas statistical heritability describes how transformed parental variables (e.g., mean IQ of parents) relate to transformed offspring variables (e.g., mean IQ of children), telling us nothing about the relative contribution of each parent's “intelligence” genes (whatever these may be) to the child's IQ. As for “intelligence,” it is tautologically defined as being “that” which intelligence tests measure (p. 58), providing little scope for external validation of the construct. The author suggests we replace “intelligence” with “adaptability” as a more quantifiable and better defined proxy of human achievement (p. 61), converging with mainstream approaches declaring the many benefits of psychological flexibility in learning contexts (Asikainen et al., 2018). The chapter provides excellent talking points for the always-popular coffee table topics of heredity and intelligence.

The remainder of the book focuses on Economics and related areas, which are shown to be also rife with fuzzily defined concepts (how do we measure “affluence”?—p. 73; when is a market “efficient”?—p. 116) mired under a guise of mathematical formalism. Of note is a fascinating critique of the highly-cited Prospect Theory (PT), which led its authors to a Nobel Prize in Economics. JS is unimpressed, chastising PT's authors for re-framing operations under labels like “combination, segregation, isolation, coalescing” without actually explaining what these processes involved. Furthermore, the author illustrates how nearly a third of all subjects tested under PT produced results directly contradicting PT (p. 107), highlighting how even eminent Nobel prize winners are not impervious to scientific error.

The book concludes with a discussion on why outcome-focused (functional) and causal (mechanistic) models are equally essential for scientific understanding, for while a good functional model can produce predictable outcomes, the lack of any constraining causal analyses can obviate the very error-detection mechanisms that shields a scientist from erroneous inferences and inept theorizing (e.g., Heebner, 1891; for more recent examples, see Burgos, 2003).

The author paints a worrying picture of academia, where motions of “doing science” and the production of publications are incentivized over actual scientific understanding. Fortunately, JS is more then up to the task of providing a much-needed counter-balance, producing a text that should be required reading in any introductory class on research methods. *SciM* is a welcome addition to the literature on scientific research methods and is highly recommended for all students of behavior.

## Author contributions

The author confirms being the sole contributor of this work and has approved it for publication.

### Conflict of interest statement

The author declares that the research was conducted in the absence of any commercial or financial relationships that could be construed as a potential conflict of interest.
